# A swarm-optimizer-assisted simulation and prediction model for emerging infectious diseases based on SEIR

**DOI:** 10.1007/s40747-022-00908-1

**Published:** 2022-11-16

**Authors:** Xuan-Li Shi, Feng-Feng Wei, Wei-Neng Chen

**Affiliations:** School of Computer Science and Engineering, South China University of Technology, Guangzhou, 510006 China

**Keywords:** Emerging infectious diseases, Epidemic mechanism, Swarm optimizer

## Abstract

Mechanism-driven models based on transmission dynamics and statistic models driven by public health data are two main methods for simulating and predicting emerging infectious diseases. In this paper, we intend to combine these two methods to develop a more comprehensive model for the simulation and prediction of emerging infectious diseases. First, we combine a standard epidemic dynamic, the susceptible–exposed–infected–recovered (SEIR) model with population migration. This model can provide a biological spread process for emerging infectious diseases. Second, to determine suitable parameters for the model, we propose a data-driven approach, in which the public health data and population migration data are assembled. Moreover, an objective function is defined to minimize the error based on these data. Third, based on the proposed model, we further develop a swarm-optimizer-assisted simulation and prediction method, which contains two modules. In the first module, we use a level-based learning swarm optimizer to optimize the parameters required in the epidemic mechanism. In the second module, the optimized parameters are used to predicate the spread of emerging infectious diseases. Finally, various experiments are conducted to validate the effectiveness of the proposed model and method.

## Introduction

Since 1980, more than 30 emerging infectious diseases (EIDs) have appeared in the world, such as SARS, COVID-19, and so on [1]. In particular, up to Dec. 2021, more than 260 million people were infected by COVID-19 and about 5.4 million people died of it, according to the report from the World Health Organization (WHO) [2]. Moreover, due to the interconnection among humans, animals, and environments, it is hard to completely stop the occurrence of EIDs in the future [3]. What humans can do is to take measures to control and prevent the spread of EIDs. Therefore, it is significant to discover EIDs as early as possible, simulate and predict the spread of EIDs, and control the spread at an early stage.

The simulation and prediction of infectious diseases are research hotspots in the field of public health. During the past decades, researchers have developed some mathematical mechanisms to uncover the general principles and spread process of infectious diseases [4]. Among them, the susceptible–infectious–recovered (SIR) model [5] and its extended models are most recognized [6–11]. In 1927, Kermack and Mckendrick developed the SIR model to investigate the Bubonic plague propagated in London [5]. The idea of the SIR model is to use a dynamic system to track the transmission of the virus among disparate nodes in a network. Each node represents one of the three states (S, I, R) in this system.

Following the work of Kermack and Mckendrick, many extensions and variants of SIR have been proposed to simulate epidemic spread. The susceptible–exposed–infected–recovered (SEIR) model [6, 7] is a famous extension of the SIR model. Li and Muldowney [6] introduced an exposed stage E, in which people are infected but unconscious about that. They studied the SEIR model with nonlinear incidence rates in epidemiology. Besides, the susceptible–exposed–infected–vaccinated (SEIV) model is another famous extension of the SIR model [8, 9]. Cai et al. [8] investigated the SEIV model with a nonlinear incidence rate, which exhibits the disease-free equilibrium and the endemic equilibrium. The extensions of the SIR model are gained not only by adding epidemic characteristics but also by changing or removing the original epidemic characteristics. The susceptible–infected–susceptible (SIS) model is yielded by deleting the recovered state and reusing one more susceptible state [10, 11]. In [10], the authors presented a sufficient condition for asymptotic stability of the healthy equilibrium and proved it based on the SIS model.

Based on these commonly used epidemic mathematical mechanisms, some researchers proposed the improved versions to simulate EIDs and used numerical methods to optimize the parameters of mechanisms [12–16]. According to the transmission features, prevention and control strategies, Zu et al. [12] constructed a compartmental model for the EID, named susceptible–exposed–infectious–suspected–confirmed–recovered (SEISCR), and used the least square method and Markov Chain Monte Carlo method to simulate the parameters. Considering the spread of the EID in Northern Italy, Jose et al*.* [13] performed the analysis of parameters and the initial condition of a deterministic SEIR model, which is solved by a forward Euler finite-difference scheme. Rezapour et al.[14] use the Caputo fractional derivative to provide a SEIR model for the EID, and then they investigated the feasibility region and stability of equilibrium points. Although there are some studies on various improved models, they mainly focus on the improvement and analysis of epidemic mechanisms. The numerical optimization methods to optimize the parameters of epidemic mechanisms have not been studied in depth.

Apart from mechanism-driven studies, some researchers considered data-driven methods to simulate and predicate the spread of EIDs. Different from mechanisms-driven methods, data-driven methods directly discover internal relationship from the initial data and automatically build model for problems. Machine learning is a commonly used data-driven method [17–21]. Based on the neural network, Wieczorek et al. [17] used a deep architecture, NAdam training model, to forecast the spread of the EID. This method can result in 99% accuracy in some cases. Rustam et al. [18] applied four machine learning forecasting models, linear regression, support vector machine, least absolute shrinkage and selection operator, and exponential smoothing in their research to predicate the spread of the EID. Hybridizing a 1-D discrete wavelet transform, Hazarika et al*.* [20] considered the random functional link network to improve the accuracy over the long-term forecast for the EID. Moreover, time series method is another major used data-driven method [22–25]. Based on two-piece scale mixture normal distributions, Maleki et al. [23] used autoregressive time series models to forecast the time series data of the EID. Many traditional symmetric/asymmetric and light/heavy tailed autoregressive models are involved in Mohsen’s model.

Although various mechanism-driven and data-driven methods have been proposed, there remain some limitations in the existing studies. On the one hand, mechanisms, particularly complex mechanisms, are heavily rely on prior knowledge and assumptions, but sometimes the simulation result of mechanisms may be wrong [4]. Moreover, if more practical factors are considered, epidemic mechanisms would become much more complex with a lot of parameters. It is difficult to determine the proper values of these parameters. Consequently, the accuracy of the mechanism-driven method would be affected. On the other hand, though data-driven methods can track the spreading tendency of EIDs approximatively, they have poor interpretability. In other words, data-driven methods are limited in elucidating the spread and persistence principles of EIDs. Moreover, at the early stage of EIDs, collecting high-quality data is difficult, and the low-quality collected data bring uncertain influence on data methods.

Fortunately, the characteristics of mechanism-driven and data-driven methods are complementary. Containing the features of EIDs in data, data-driven methods can help epidemic mechanisms reduce the requirement of assumptions and parameters. While revealing the spread process of EIDs, mechanism-driven methods can help data-driven method improve the interpretability and reduce the uncertainty caused by bad data. Therefore, it is promising to integrate the mechanism-driven method and the data-driven method to explore the study of EIDs.

Few studies have used both mechanism-driven and data-driven methods [26, 27]. Yang et al. [26] derived the epidemic curve from the SEIR model, and used an artificial intelligence approach to predict the epidemic. Feng et al*.* [27] used the SEIR model to simulate and predict the epidemic spread trend in Wuhan and used the data-driven method (LSTM) in non-Wuhan areas. However, they just use the mechanism-driven method and the data-driven method to separately simulate and predict the spread of the EID in different regions. The characteristics of these two kinds of models are not combined. Moreover, although mechanism-driven methods also require the actual data [12–16], the actual data are only used as target in evaluation function or objective function.

Therefore, to alleviate the complexity and lower accuracy of the mechanism-driven method and lower interpretability and high data dependency of the data-driven method, we focus on combining mechanism-driven and data-driven methods. We directly apply the initial data to the epidemic mechanism, revealing the transmission characteristics that are not discovered by the epidemic mechanism. Thereby the actual data drives the simulation of the epidemic spread and the epidemic mechanism provides the biological spread process of the EID. The major contributions of this paper are as follows.

1. Considering the significant influence of population migration on the EID spread, we combine the population migration into the SEIR model and then build a discrete form model of the EID with the population migration.
2. Based on the above model, we propose a data-driven parameter optimization approach. In this approach, aggregating the actual data of the EID and the population migration, we model the process of determining model parameters as a data-driven optimization process. Meanwhile, an objective function is constructed in this approach to minimize the error of the simulated data and the actual data. So far, an epidemic mechanism-driven and data-driven model (EMDE) is constructed. It is consisting of an improved epidemic mechanism with population migration and a data-driven parameter optimization approach.
3. On the basis of EMDE, we propose a swarm-optimizer-assisted simulation and prediction method, which contains two modules, the simulation module and the prediction module. In the simulation module, a level-based learning optimizer is used to search the best parameter set though EMDE. Combining the optimal parameter set, the prediction module uses the improved epidemic model with population migration to predicate the EID spread in the next few days. An average strategy driven by the actual data is used to predicate the population migration.

The rest of this paper is organized as follows. We describe the background information of the particle swarm optimizer, population migration, and a traditional epidemic mechanism in “Backgrounds”. Then, the process of building the improved model is provided in “The epidemic mechanism-driven and data-driven model”. Afterward, we describe how to simulate and predicate the EID spread in “Swarm-optimizer-assisted simulation and predication method”. To certify the effectiveness, experiments are conducted in “Experiments”. Finally, we summarize this article in “Conclusions”.

## Background

Since the problem considered in this paper is based on epidemic mechanism, background about the traditional SEIR model is introduced [6, 28, 29]. Moreover, with the human interconnection increasing, the influence of human activities on EIDs is unignorable, especially the population migration [3]. Furthermore, to give a better description of our methods, we explain some basic techniques about particles swarm optimization (PSO) [30].

### Traditional SEIR epidemic mechanism

**Fig. 1:**
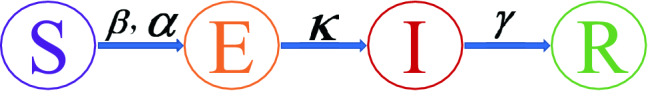
State transition process of SEIR

Although many effective epidemic mechanisms have been proposed, we prefer the SEIR model for its appropriate number of parameters and more in line with the transmission characteristics of most EIDs at the early stage. The SEIR model simulates the spread of the epidemic based on infection states and their connection, where four abbreviations represent four different states drawn in Fig. 1. In the SEIR model, the susceptible state (S) means that people are in a case, where they have low immunity to the virus and are easy to be infected. The exposed state (E) means that people are infected but are not detected. Therefore, they unintentionally expose the virus to healthy people. Analogously, the infected state (I) means that people are infected. The recovered state (R) means that the recovered people will never be infected again. Then, we give a traditional formal of the SEIR model in Eq. (1), and summarize the explanation of parameters in Table 1:

1 $$ \left\{ \begin{gathered} \Delta S = - \frac{\beta S(t)I(t)}{{N(t{)}}} - \frac{\alpha S(t)E(t)}{{N(t{)}}} \hfill \\ \Delta E = \frac{\beta S(t)I(t)}{{N(t{)}}} + \frac{\alpha S(t)E(t)}{{N(t{)}}} - \kappa E(t) \hfill \\ \Delta I = \kappa E(t) - \gamma I(t) \hfill \\ \Delta R = \gamma I(t) \hfill \\ \end{gathered} \right., $$

2 $$ N(t) = S(t) + E(t) + I(t) + R(t). $$

**Table 1:** Parameters of SEIR

Variable	Explanation
*S*(*t*)	The number of susceptible people at time *t*
*E*(*t*)	The number of exposed people at time *t*
*I*(*t*)	The number of infected people at time *t*
*R*(*t*)	The number of recovered people at time *t*
*N*(*t*)	The total number of people involved in the EID at time *t*
*β*	The probability of a susceptible person to be affected by the infected and then transform into the exposed
*α*	The probability of a susceptible person to be affected by the exposed and then transform into the exposed
*κ*	The probability of an exposed person transforms into being infected
*γ*	The probability of an infected person recovers

### Population migration

With the rapid development of society, the interconnection among humans become more and more frequent. Especially as transportation facilities become much more convenient, the large population movement is a significant difference between the spread of EIDs and past traditional viruses [3, 31–33]. For example, more than 5 million people have left Wuhan, one of China’s transportation hubs, during the outbreak of COVID-19 [34]. In [35], Du et al. considered the risk for the transportation of COVID-19 from Wuhan to other cities. They expected that, before the quarantine, the infected risk of 130 cities is more than 50%, and the infected risk of the 4 largest metropolitan areas is up to 99%. Therefore, it is promising to combine population migration with epidemic mechanisms. Briefly, the population migration among *K* different cities can be abstracted as a dynamic matrix Eq. (3):

3 $$ M(t) = \left( {\begin{array}{*{20}c} {m_{1,1} (t)} & \ldots & {m_{1,K} (t)} \\ \vdots & \ddots & \vdots \\ {m_{K,1} (t)} & \cdots & {a_{K,K} (t)} \\ \end{array} } \right), $$

where m_*i,j*_(*t*) is the number of people who migrated from the *i*^th^ city to the *j*^th^ city at time *t*, and *K* is the number of cities. According to Eq. (3), the number of people flow out and in the *i*^th^ city can be represented by

4 $$ m_{i}^{out} (t) = \sum\limits_{j = 1}^{K} {m_{i,j} (t)} , $$

5 $$ m_{t}^{in} (t) = \sum\limits_{j = 1}^{K} {m_{j,i} (t)} , $$

where $$ m_{t}^{out} (t)$$ is the total number of people moving out from the *i*^th^ city at time *t*, while $$ m_{t}^{in} (t)$$ is the total number of people moving into the *i*^th^ city at time *t.*

### Particle swarm optimizer

Inspired by the intelligent behaviors of social animals, Eberhart and Kennedy [30] proposed PSO, where a swarm of particles traverses the whole solution space to find the global optimum. PSO is a widely used evolutionary computation algorithm [36]. In PSO, each particle presents a candidate solution in the swarm. By some learning strategies, particles learn from other particles to guide themselves to find the optimum. With the good exploration and easy implementation, PSO has been extensively studied and applied in many optimization problems [37–40]. For example, Zhao et al. [37] proposed a swarm-based stochastic optimization policy to control the spread of the epidemic and allocate the resource efficiently.

In this paper, a level-based learning swarm optimization (LLSO) algorithm is considered, which is first proposed in [41] for the large-scale problem. Based on PSO, two novel strategies are proposed in LLSO, including the level-based learning strategy and exemplar selection strategy. In the level-based learning strategy, particles are sorted according to fitness and then divided into several levels. Better particles belong to higher levels with small indexes. In the exemplar selection strategy, particles are allowed to randomly pick up two particles as exemplars, respectively, from two higher levels. Particularly, particles in the second level only learn from the first level, and the particles in the first level reserve themselves. The whole process of the LLSO algorithm is shown in Algorithm 1.
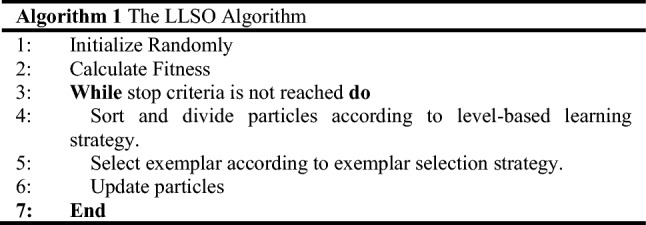


## The epidemic mechanism-driven and data-driven model

The main work of this paper is to solve a simulation and prediction problem for the EID, under the increasing effect of population migration. To address this issue, we combine a standard SEIR epidemic model with the migration population to reveal the EID spread process. However, parameter values in the mechanism are different in different EIDs. To determine the parameters of this mechanism, we further obtain the public health data and population migration. Based on these two sets of data, we abstract the parameter solving process as a data-driven process and define a data-driven objective function. Besides, the spread of the EID in each city is calculated separately, since the spread characteristics for the EID and population migration vary in different regions.

### The epidemic mechanism with population migration

Without restrictions on traffic at the early stage of EIDs, people of different epidemic states may flow out or into cities, which increases the spread of EIDs. In other words, people in high-risk cities many carry the virus to uninfected cities. Moreover, with the high-speed development of traffic, people can travel across many areas in 1 day, resulting in a faster spread speed of EIDs. Consequently, based on the studies of other researchers [31, 42], we consider combining the population migration with the epidemic mechanism in the following formulations. First, a matrix *y*_*i*_(*t*) is constructed to present the number of people in different states for the *i*^th^ city at time *t*:

6 $$ y_{i} (t) = \left[ \begin{gathered} S_{i} (t) \hfill \\ E_{i} (t) \hfill \\ I_{i} (t) \hfill \\ R_{i} (t) \hfill \\ \end{gathered} \right]. $$

Since there are well-protected people and the total population of a city is too large, not all the people of a city are involved in the EID. Therefore, *N*_*i*_(*t*) is not equal to the total population of a city *Z*_*i*_. In other words, only a part of migration people may be susceptible, exposed, infected, or recovered. We summarize the population migration of different states for the *i*^th^ city in Eqs. (7) and (8):

7 $$ y_{i}^{out} (t) = \frac{{m_{i}^{out} (t)}}{{Z_{i} }}*y_{i} (t), $$

8 $$ y_{i}^{in} (t) = \sum\limits_{j = 1}^{K} {\frac{{m_{j,i}^{out} (t)}}{{Z_{j} }}*y_{j} (t)} . $$

After calculating population migration of each city, we integrate the above equations with Eq. (1), and obtain the change value of *y*_*i*_(*t*):

9 $$ \Delta y_{i} (t) = \left[ \begin{gathered} \Delta S_{i} (t) \hfill \\ \Delta E_{i} (t) \hfill \\ \Delta I_{i} (t) \hfill \\ \Delta R_{i} (t) \hfill \\ \end{gathered} \right] + y_{i}^{in} (t) - y_{i}^{out} (t). $$

Finally, the discrete form of the EID combined with population migration is obtained in the following:

10 $$ y_{i} (t + 1) = y_{i} (t) + \Delta y_{i} (t). $$

### Data-driven parameter optimization

Based on the past experience, the epidemic mechanism may be useful to reveal potential the EID spread process. However, it may also ignore some emerging characteristics. The actual data can narrow the gap between the epidemic mechanism and the EID by reflecting uncaptured information. Moreover, although the improved model describes the spread of the EID with population migration, parameters in the model are unclear. To track the changeable characteristics of the EID, a data-driven parameter optimization approach is developed. It uses the actual data (**Φ**)**,** which assembles the public health data and population migration data as follows:

11 $$ {{\varvec{\Phi}}} = \{ \widetilde{I}_{i} (t),\widetilde{R}_{i} (t),\widetilde{M}_{i} (t)|{\text{for }}t = 1,2,...,T{1}, \, i = 1,2,...,K\} . $$

Variables with a wavy line above represent the actual data. The parameter set *θ*_*i*_ for each city constitute **Θ**, which contains the parameter set of all cities*:*

12 $$ \theta_{i} = [\beta_{i} ,\alpha_{i} ,\kappa_{i} ,\gamma_{i} ,S1_{i} ,E1_{i} ]. $$

Apart from the transform probabilities among different epidemic states, the number of susceptible (*S*1_*i*_) and exposed people (*E*1_*i*_) on the first day of the EID also need to be optimized. Because there is no explicit principle to determine the susceptible population. Meanwhile, governments and hospitals are hard to collect the number of exposed people.

The improved mechanism, Eq. (10), cooperates with **Θ** and **Φ** to model the process of determining model parameters as a data-driven optimization process. We define this process in the following. *A*_*i*_ is the coefficient matrix and is defined as Eq. (13):

13 $$ A_{i} = \left[ {\begin{array}{*{20}c} 0 & 0 & {0} & {0} \\ {0} & {\kappa_{{\text{i}}} } & {0} & {0} \\ {0} & {0} & {{ - }\gamma_{{\text{i}}} } & {0} \\ {0} & {0} & {\gamma_{{\text{i}}} } & {0} \\ \end{array} } \right]. $$

Moreover, *F*_*i*_(*t*) is the constant term, and vary with migration population and the spread of the EID:

14 $$ {\text{F}}_{i} (t) = \left[ {\begin{array}{*{20}c} {\frac{{ - (\beta_{i} \widetilde{I}_{{\text{i}}} (t) + \alpha_{i} E_{i} (t))S_{i} (t)}}{{N_{i} (t)}}} \\ {\frac{{(\beta_{i} \widetilde{I}_{{\text{i}}} (t) + \alpha_{i} E_{i} (t))S_{i} (t)}}{{N_{i} (t)}}} \\ 0 \\ 0 \\ \end{array} } \right] + \widetilde{y}_{i}^{in} (t) - \widetilde{y}_{i}^{out} (t). $$

Thus, based on the coefficient term and constant term, Eq. (10) can be presented as

15 $$ {\text{y}}_{{\text{i}}} (t + 1) = A_{i} y_{i} (t) + F_{i} (t). $$

Particularly, when *t* = 1

16 $$ y_{i} (1) = \left[ {\begin{array}{*{20}c} {S1_{i} } \\ {E1_{i} } \\ {\widetilde{I}_{i} (1)} \\ {\widetilde{R}_{i} (1)} \\ \end{array} } \right]. $$

And then, the spread of the EID over *K* cities is

17 $$ Y(t) = \sum\limits_{i = 1}^{K} {y_{i} (t)} . $$

18 $$ I(t) = \sum\limits_{i = 1}^{K} {y_{i} (t)} [3] = Y(t)[3]. $$

In addition, *I*(*t*) is the third element in *Y*(*t*), representing the total infected population over *K* cities on the *t*^th^ day. Furthermore, considering the number of infected people and recovered people can be required from **Φ**, the EID spread of the *t*^th^ day can be represented by

19 $$ y_{i} (t) = \left[ {\begin{array}{*{20}c} {{\text{y}}_{i} (t)[1]} \\ {{\text{y}}_{i} (t)[2]} \\ {\widetilde{I}_{i} (t)} \\ {\widetilde{R}_{i} (t)} \\ \end{array} } \right], $$

where *y*_*i*_(*t*) [1] is the simulated number of susceptible people and *y*_*i*_(*t*) [2] is the simulated number of exposed people. Finally, based on the above equations, we construct the objective function to minimize the error between the actual infected population and the simulated for *K* cities over *T*1 days. In other words, the parameters are optimized to obtain a minimum gap. *w* is a scale factor:

20 $$ f(I_{i} (t);{{\varvec{\Theta}}},{{\varvec{\Phi}}}) = {\text{MIN}}\sum\limits_{t = 1}^{T1} {|\widetilde{I}(t) - I(t)|^{w} } . $$

So far, we finish the construction of EMDE. On the one hand, we combine the SEIR model with migration population and provide a linear expression form for mathematical convenience. The epidemic mechanism can take off the EID’s spread process and give a better interpretation to help humans resist the EID. On the other hand, the actual data of the infected population, the recovered population, and population migration are used to drive the optimization of parameters. the actual data can revise the epidemic mechanism deviating from the truth.

In general, EMDE combines the mechanism-driven method and the data-driven method to simulate and predict the spread of the EID. On the one hand, with the epidemic mechanism with population migration, EMDE can reveal the biological spread process of the EID and reduce the overfocus on the data. On the other hand, with the data-driven parameter optimization approach, EMDE can timely revise the gap between the actual epidemic spread and the simulated epidemic spread. The actual data (**Φ**) used in the approach can reflect some uncaptured information by the epidemic mechanism. If the simulated epidemic spread deviates from the actual spread, EMDE can timely revise this deviation by the proposed data-driven approach. Therefore, EMDE combines the advantages of epidemic mechanism-driven and data-driven methods. Meanwhile, these two methods can complement each other’s disadvantages in EMDE.

## Swarm-optimizer-assisted simulation and predication method

Subsequently, a swarm-optimizer-assisted simulation and prediction method is introduced, which is consisted of two modules. In the first simulation module, an improved swarm optimizer is used to assist the parameter optimization, and the objective function, Eq. (20), is adopted to calculate the fitness of particles. In the second prediction module, the optimized parameters and simulated data from the simulation module are used to predicate the spread of the EID.

### Swarm-optimizer-assisted simulation module

#### Module description

**Fig. 2:**
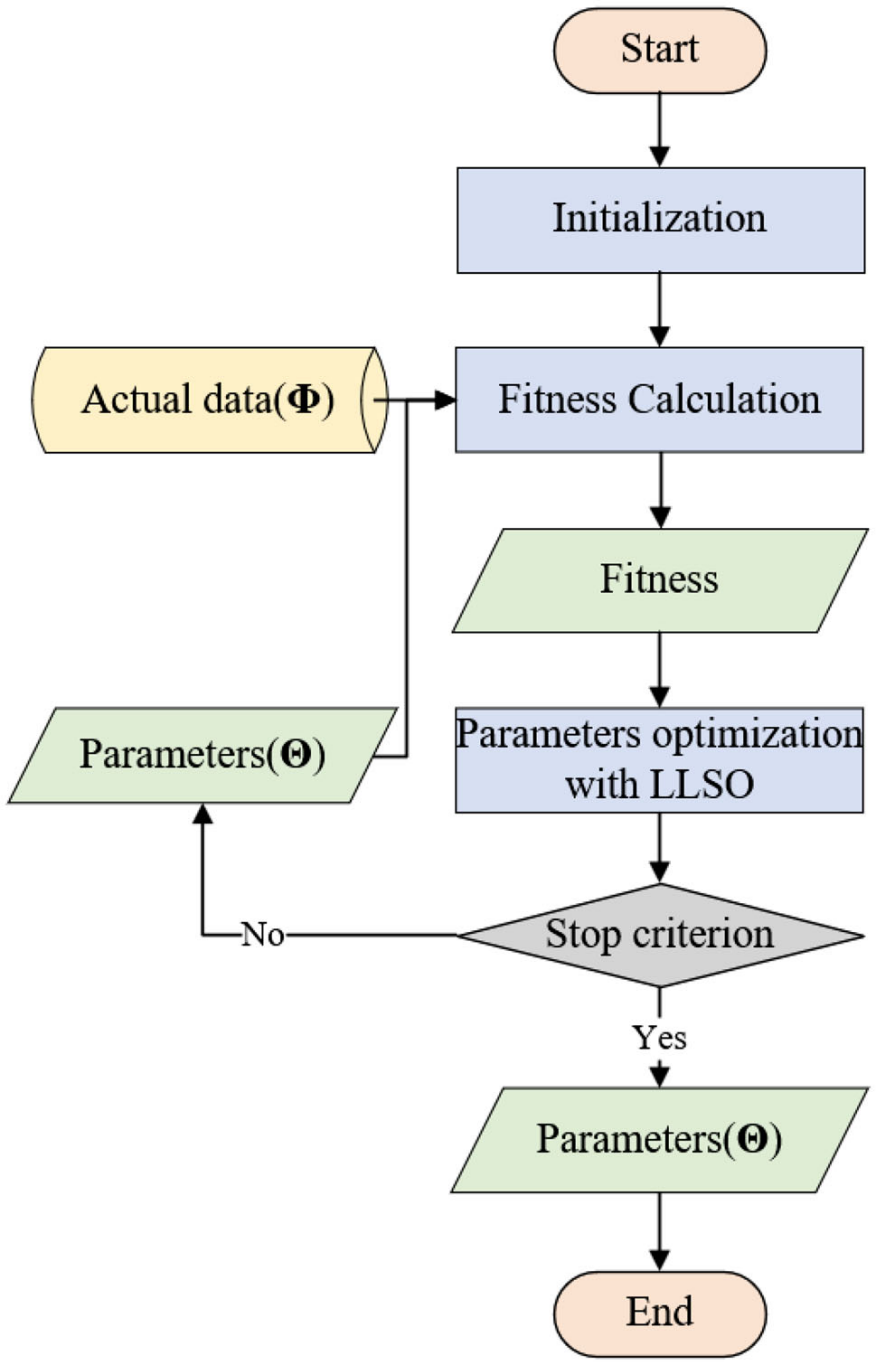
Flow chart of the simulation module

The main idea of the simulation module is to help EMDE explore the optimal parameters. For this purpose, three submodules are developed and the flowchart is drawn in Fig. 2. First, in the initialization submodule, suitable expressions of solutions are generated and initialized according to the range of parameters. Every solution is a combination of parameters represented by a particle. Second, with the generated parameter’s value, we can use mathematical formulas defined in Eq. (10) to simulate the spread of the EID in the fitness calculation submodule. This submodule outputs the result calculated by Eq. (20) of each solution as fitness to the next submodule. Third, according to the learning strategies of LLSO, all particles update themselves to search the optimum. If the stop criterion is satisfied, the simulation module outputs the best combination of parameters and is terminated. Otherwise, all parameter combinations are delivered to the fitness calculation submodule. In general, the stop criterion is related to the number of evaluations.

#### Initialization

First of all, we construct the position of particles to represent solutions in the initialization submodule. Each solution is a combination of parameters for *K* cities. Each city has |*θ*_*i*_| parameters, where |*θ*_*i*_| is the size of *θ*_*i*_. Thus, the dimension of a solution is K*|*θ*_*i*_|, *D* = K*|*θ*_*i*_|. Considering the convenient transportation nowadays, the interconnection between cities is frequent, and *K* is generally a large number. Therefore, the parameter optimization has a high-dimensional search space. To better optimize the large-scale problem, a recently proposed large-scale optimizer, LLSO is adopted in the third submodule. Besides, because the range of parameters are different, we order them according to their range. The position of the *n*^th^ particle can be presented by

21 $$ x_{n} = [\beta_{1} ,\alpha_{1} ,\kappa_{1} ,\gamma_{1} ,...,\beta_{K} ,\alpha_{K} ,\kappa_{K} ,\gamma_{K} ,S1_{1} ,E1_{1} ,...,S1_{K} ,E1_{K} ]. $$

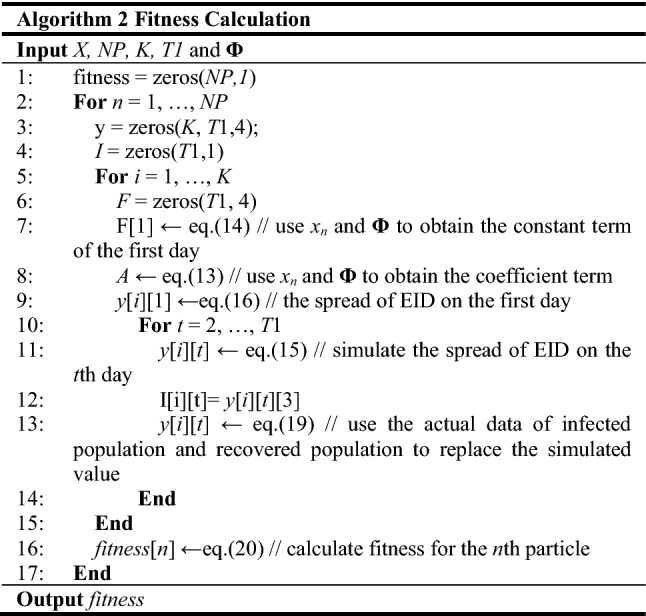


After constructing the position expression, we randomly generate the value for each particle. All transform probabilities among different epidemic states are within [0,1] and the range of *S*_*i*_(*t*) and *E*_*i*_(_t_) are within [0, *Z*_*i*_]. For values out of range, they are assigned the corresponding upper or lower. The velocity of the *n*^th^ particle is represented by *v*_*n*_ and also initialized by the above method. All positions of particles form *X*. All velocities of particles form *V*.

#### Fitness calculation

After initializing the positions of particles, the fitness of each particle is calculated in this submodule. The fitness represents the distance from the optimum position to the particle’s position. In this paper, particles with smaller fitness have more useful information to explore the optimum. Meanwhile, digging the promising information to guide particles which are away from the optimum, can accelerate the convergence of the whole swarm. Thus, it is significant to find out useful information from better particles in Algorithm 2.

First, the fitness of all particles is set to be zero. A loop is started to calculate the fitness for each particle in lines 1–2. Specifically, function zeros(*number1*, *number2*) means creating an all zeros matrix with two dimensions, where the first dimension size is *number1* and the second dimension size is *number2*. Entering the loop, the simulated data is initialized to zero and the process goes to the inner loop in lines 3–5. Next, for each city, we can use the parameters’ value in *x*_*n*_ and the corresponding equation to initialize *F*_*i*_, *A*_*i*_, and *y*_*i*_, at the first day. Then, the spread of EID over *T*1 days for the *i*^th^ city can be simulated in lines 10–14. After simulating the spread of the EID for *K* cities over *T*1 days, the fitness of one particle is calculated in line 16. By the above process, we can obtain the fitness of all particles.

#### Parameters optimization with a level-based learning swarm optimizer

To obtain the optimum value of the parameters, we use a level-based learning swarm optimizer (LLSO) in this submodule. As mentioned in the background of the LLSO algorithm, the level-based learning strategy and the exemplar selection strategy are constructed to evolute particles. Based on these two strategies, particles are updated by

22 $$ \left\{ \begin{gathered} v_{i,j}^{d} \leftarrow r_{1} v_{i,j}^{d} + r_{2} (x_{{rl_{{_{1} }} ,k_{1} }}^{d} - x_{i,j}^{d} ) + \varphi r_{3} (x_{{rl_{2} ,k_{2} }}^{d} - x_{i,j}^{d} ) \hfill \\ x_{i,j}^{d} \leftarrow x_{i,j}^{d} + v_{i,j}^{d} \hfill \\ \end{gathered} \right., $$

where the LLSO algorithm allows each particle in level *L*_*i*_ to learn from two particles $x_{{rl_{{_{1} }} ,k_{1} }}^{d}$ and $x_{{rl_{{_{2} }} ,k_{2} }}^{d}$. They are randomly selected from two different higher levels $L_{{rl_{{_{1} }}}}$ and $L_{{rl_{{_{2} }}}}$. *rl*_1_ and *rl*_2_ are two numbers that are randomly selected from [1, i-1], respectively, *k*_1_ and *k*_2_ are random integers selected from [1, *NP*/*NL*]. *r*_1_, *r*_2,_ and *r*_3_ are three random numbers within [0,1], and *φ* is also within [0,1] to control the influence of the second exemplar.

Thus, by Eq. (22), particles can update themselves by learning from better particles to close in the optimum. In other words, as well as particles update, parameters are optimized to minimize the gap between the simulated value and the actual data.
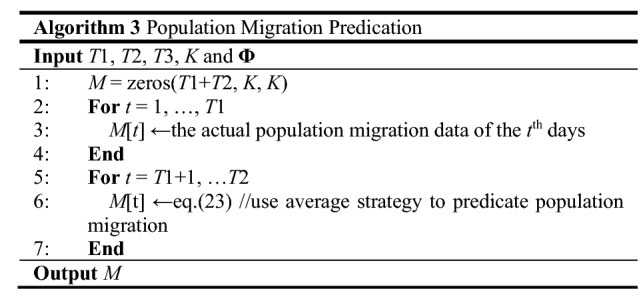


### Prediction module

#### Module description

**Fig. 3:**
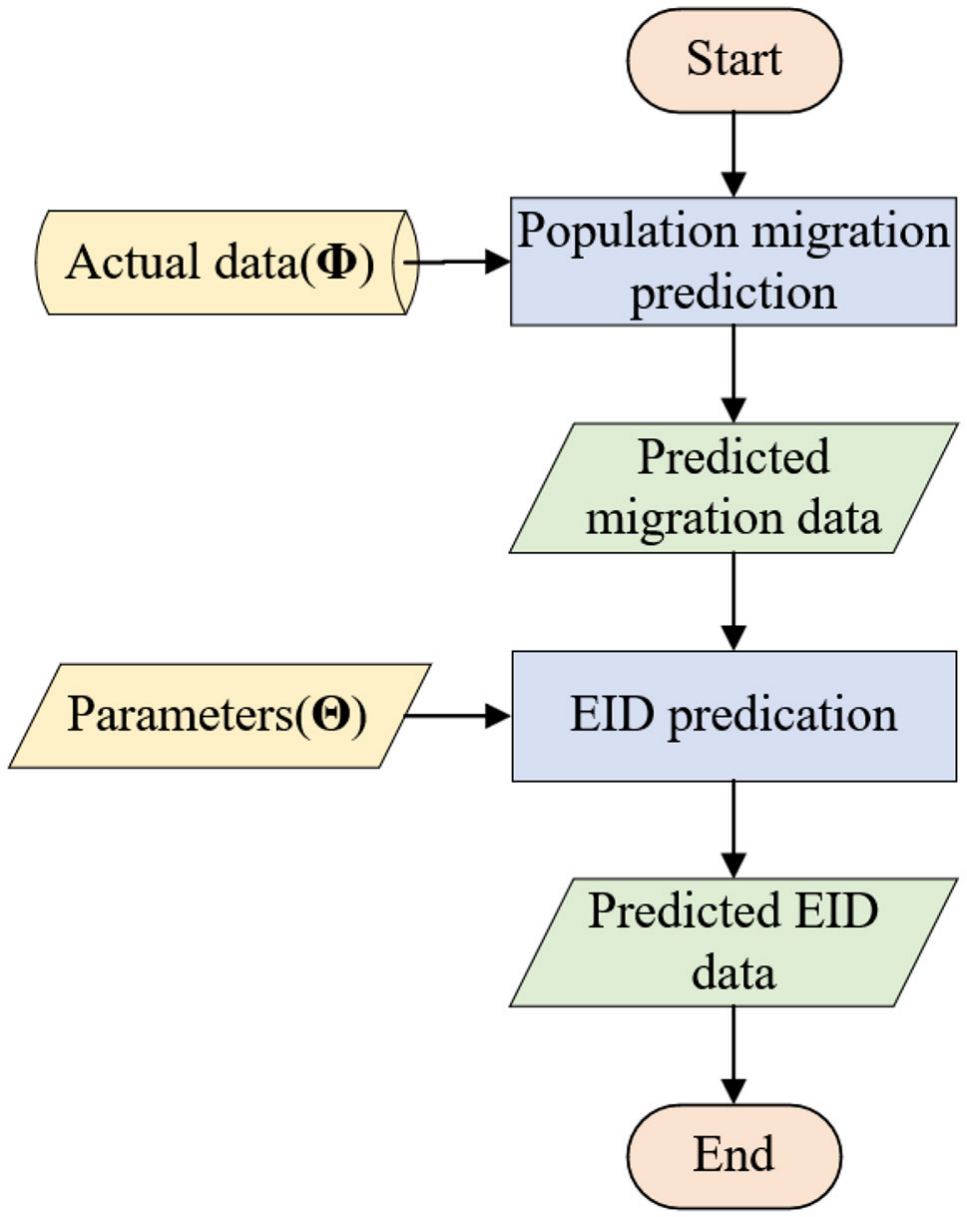
Flow chart of the prediction module

Based on the output parameters from the simulation module and the epidemic mechanism, we can predicate the spread of EID in the next *T*2 days. The prediction module contains two submodules, as shown in Fig. 3. First of all, in the population migration prediction submodule, a simple strategy is used to predicate the population migration in the next *T*2 days. This submodule outputs the predicated population migration data to the next submodule. Second, in the EID predication submodule, we can use Eq. (10) to calculate the spread of EID in the next *T*2 days.

#### Population migration predication

What we focus on in this paper is the spread of the EID. However, there is no population migration mechanism to predicate the migration between *K* cities. Thus, an average strategy is used to predicate population migration. Due to the population migration in approach days is similar, we use the average population migration in the previous *T*3 days to represent population migration in the next day. In other words

23 $$ M{(}t) = \left(\sum\limits_{p = 1}^{T3} {M(t - p)} \right)/T{\text{3, for all }}t{ = }T{1 + 1,}...{,}T{1 + }T{2}{\text{.}} $$

We summarize this process in Algorithm 3. First of all, we initialize the population migration matrix (*M*) of *T*1 + *T*2 to be zeros. Then, in lines 2–3, the actual data of population migration for *T*1 days are filled into *M*. Finally, according to Eq. (23), we can predicate the population migration in the next *T*2 days.
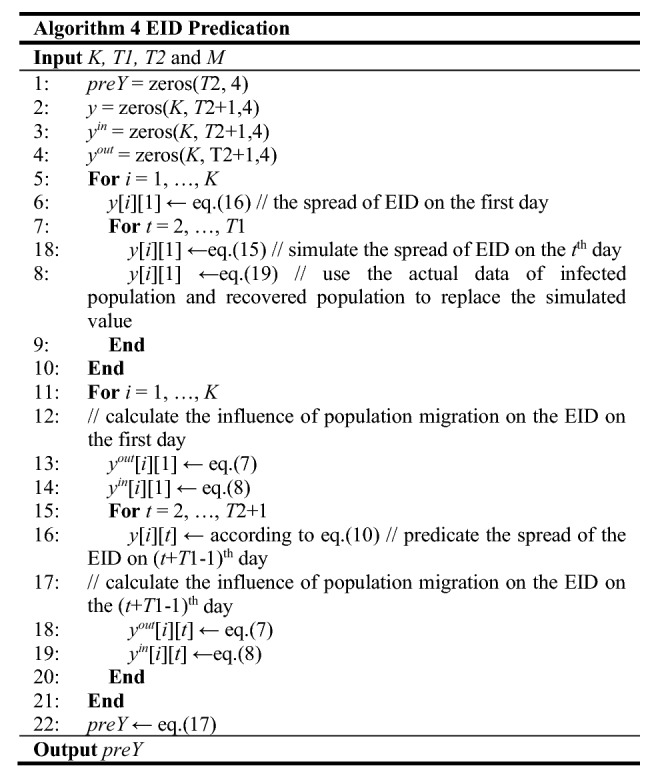


#### EID predication

According to Eq. (10), the EID spread situation of the *T*1th day, *y*_*i*_(*T*1), is critical to the EID prediction in next the *T*2 days. However, only the number of infected people and the number of recovered people can be required from the actual data. Thus, we simulate the spread of EID until the *T*1th day to obtain *S*_*i*_(*T*1) and *E*_*i*_(*T*1) for all cities. Moreover, with explicit *y*_*i*_(*T*1), the influence of EID in the specified city on other cities, though population migration, can be captured. All process of EID predication is summarized in Algorithm 4. After initializing variables in lines 1–4, the EID spread of *K* cities over *T*1 days is simulated in lines 5–11. And then, the process enters a loop to predicate the EID for each city in line 12. At the beginning of the loop, we calculate $y_{i}^{out} (T1)$ and $y_{i}^{in} (T1)$. Then, the EID predication is implemented in lines 15–16.

### Complexity analysis

In this section, we make a complexity analysis of the proposed method from three parts. First, the time complexity of the fitness calculation submodule in Algorithm 1 is *O*(*NP* × *K* × *T*1). Second, according to the complexity analysis of [41], the LLSO algorithm takes *O*(*NP* × *D* + *NP* × log(*NP*)) without considering the time of function evaluations. Thus, the time complexity of the parameter optimization submodule is *O*(*NP* × *D* + *NP* × log(*NP*)). Third, from Algorithm 4, the time complexity of the EID prediction submodule is *O*(*K* × *T*1 + *K* × *T*2). We ignore the time complexity analysis of other submodules, since their complexity is smaller compared with the above parts. Therefore, the total time complexity of the proposed method is *O*(*NP* × (*K* × *T*1 + *D*) + *NP* × log(*NP*)).

## Experiments

To certify the effectiveness of the proposed model (EMDE) and method, four comparison experiments are conducted in this section. In the first experiment, EMDE is compared with three pure data-driven methods to prove its validity. In the second experiment, we further investigate the effectiveness of the data-driven parameter optimization. In the third experiment, the swarm-optimizer-assisted module in the proposed method is compared with two numerical optimization methods to optimize parameters. In the last experiment, LLSO is compared with other swarm optimizers to prove the advantage of using LLSO in our method. To be fair, all methods conducted in the second experiment use the prediction module, which is proposed in our method.

### Parameters setting

We summarize the parameters setting in this paper in the following:

1. We take the spread of COVID-19 in China as an example to conduct experiments.
2. Authoritative data of 30 cities from January 24, 2020, to March 15, 2020, are used in our experiments. These 30 cities are selected, since they have large population in China. The name of cities is shown in Table 2. *K* is the number of cities, *K* = 30.
3. The population migration data is obtained from qianxi.baidu.com/ and the data set of COVID-19 is obtained from github.com/BlankerL/DXY-COVID-19-Data.
4. Due to *D* = K*|*θ*_*i*_|, *D* is equal to 180.
5. We set the number of particles (*NP*) in a particle swarm to 500, *NP* = *500*, and set the max evaluation number to 3000*D.
6. Attentionally, *T*1 is the simulation days, *T*2 is the prediction days, and *T*3 is the days used to predicate the population migration.
7. We use the root mean square error (RMSE) as the evaluation criteria in all experiments, where *T* is the evaluated days, $\widetilde{I}(t)$ is the actual infected number of people for *K* cities, and *I*(*t*) is the simulated or predicated the number of infected people for *K* cities.
  24 $$ {\text{RMSE}} = \sqrt {\frac{1}{T}\sum\limits_{t = 1}^{T} {(\widetilde{I}(t) - I(t))^{2} } } , $$
8. For all experiments, we repeat 20 times and use the average value as result.
9. To better exhibit results, we count the w/l/t for each experiment, which represents that our method wins on *w* other methods, loses on *l* other methods and draws on *d* other methods.
10. The *p* value of each experiment is calculated through ttest2 function in Matlab. The symbols, “ + ”, “−”, and “ = ” above the *p* value, respectively, represent our method significantly better than, worse than, and equivalent to the comparison method.

**Table 2:** Name of 30 cities

Chongqing	Shenzhen	Zhengzhou
Shanghai	Changsha	Xian
Beijing	Kunming	Ganzhou
Chengdu	Fuzhou	Handan
Tianjin	Nanyang	Wenzhou
Guangzhou	Linyi	Weifang
Wuhan	Shijiazhuang	Zhoukou
Hangzhou	Haerbin	Qingdao
Nanjing	Suzhou	Xuzhou
Changchun	Baoding	Heze

#### Comparison experiment with data-driven methods

In this experiment, three data-driven models, including polynomial regression (PR), kernel ridge regression (KRR), and autoregressive integrated moving average model (ARIMA) are compared to EMDE. We directly use these data-driven methods provided by the sklearn and the statsmodels libraries in Python. Two groups of simulation experiments are conducted, where the simulated days (*T*1), respectively, are 7 days (1 week) and 21 days (3 weeks). For each simulation group, three groups of prediction experiments are implemented, where the prediction days (*T*2) are, respectively, 3 days, 7 days, and 10 days. We summarize the result in Tables 3 and .

**Table 3:** Comparison results with data-driven methods for simulation days being 7 days (*T*1 = 7)

Predicated days	Instances/Methods	1–2 weeks		2–3 weeks		3–4 weeks		4–5 weeks	
–	–	RMSE	*P* value	RMSE	*P* value	RMSE	*P* value	RMSE	*P* value
*T*2 = 0 days	EMDE	3.42E + 02	–	4.64E + 02	–	4.11E + 03	–	2.67E + 02	–
	PR	1.20E + 02	7.64E-10^−^	8.02E + 01	1.23E-36^−^	1.28E + 03	3.50E-59^−^	1.40E + 02	9.90E-32^−^
	KRR	7.56E + 02	1.25E-14^+^	2.30E + 03	1.56E-49^+^	6.09E + 03	3.39E-56^+^	6.39E + 03	1.03E-63^+^
	ARIMA	3.48E + 02	7.67E-01^=^	3.97E + 02	3.28E-22-	4.32E + 03	1.99E-37^+^	4.26E + 02	1.35E-33^+^
	*w/l/d*	1/1/1		1/2/0		2/1/0		2/1/0	
*T*2 = 3 days	EMDE	2.27E + 02	–	7.32E + 02	–	1.52E + 03	–	7.71E + 02	–
	PR	8.77E + 02	7.07E-17^+^	1.08E + 03	1.61E-29^+^	2.88E + 04	8.87E-63^+^	2.87E + 02	3.10E-18^−^
	KRR	2.44E + 03	6.95E-27^+^	7.83E + 03	2.68E-54^+^	2.06E + 04	7.43E-60^+^	1.41E + 04	1.53E-45^+^
	ARIMA	3.43E + 02	8.25E-05^+^	2.24E + 02	1.51E-32^−^	2.53E + 03	1.36E-35^+^	2.21E + 03	3.52E-27^+^
	*w/l/d*	**3/0/0**		2/1/0		**3/0/0**		2/1/0	
*T*2 = 7 days	EMDE	1.86E + 03	–	1.49E + 03	–	5.89E + 03	–	7.94E + 02	–
	PR	7.75E + 03	3.11E-24^+^	4.48E + 03	7.83E-39^+^	1.49E + 05	6.35E-68^+^	6.01E + 03	3.68E-28^+^
	KRR	5.57E + 03	1.94E-20^+^	1.26E + 04	1.15E-49^+^	2.70E + 04	3.94E-52^+^	2.19E + 04	1.10E-39^+^
	ARIMA	2.50E + 03	4.38E-07^+^	4.42E + 02	3.28E-30^−^	3.09E + 03	1.81E-35-	4.73E + 03	7.62E-26^+^
	*w/l/d*	**3/0/0**		2/1/0		2/1/0		**3/0/0**	
*T*2 = 10 days	EMDE	4.03E + 03	–	9.05E + 03	–	1.03E + 04	–	1.06E + 03	–
	PR	1.87E + 04	2.62E-29^+^	3.74E + 03	7.51E-40^−^	3.23E + 05	1.34E-70^+^	1.56E + 04	5.07E-36^+^
	KRR	8.96E + 03	2.46E-20^+^	2.27E + 04	1.24E-47^+^	3.14E + 04	2.43E-48^+^	2.79E + 04	4.49E-41^+^
	ARIMA	4.96E + 03	1.64E-07^+^	7.95E + 03	7.74E-27^+^	6.86E + 03	1.78E-33^−^	6.74E + 03	2.88E-28^+^
	*w/l/d*	**3/0/0**		2/1/0		2/1/0		**3/0/0**	

In order to highlight the effectiveness of the proposed method, we have marked some experimental results that are significantly better than the comparison algorithms in bold

First, the simulation ability of our method is competitive. In Table 3, for *T*2 = 0 days, although the simulation performance of KRR exceeds EMDE in all instances, EMDE outperforms PR in all instances. Moreover, for ARIMA, EMDE outperforms it in two instances, loses to it in one, ties with it in one. In Table 4, for *T*1 = 21 days and *T*2 = 0 days, KRR also exceeds EMDE in all instances, but EMDE only loses to ARIMA in one. Moreover, our method draws with PR. Therefore, we recognize EMDE is competitive to data-driven models in simulation ability. In other words, EMDE is comparable to some data-driven algorithms, but it cannot be compared to all data-driven algorithms.

**Table 4:** Comparison results with data-driven methods for simulation days being 21 days (*T*1 = 21)

Predicated days	Instances/Methods	1–4 weeks		2–5 weeks		3–6 weeks		4–7 weeks	
–	–	RMSE	*P* value	RMSE	*P* value	RMSE	*P* value	RMSE	*P* value
*T*2 = 0 days	EMDE	2.73E + 03	–	2.60E + 03	–	2.48E + 03	–	2.70E + 02	–
	PR	1.67E + 03	3.61E-51^−^	2.38E + 03	3.27E-46^−^	2.55E + 03	2.36E-14^+^	3.21E + 02	5.86E-11^+^
	KRR	2.13E + 03	1.58E-46^−^	1.85E + 03	3.02E-56^−^	1.63E + 03	1.35E-34^−^	1.90E + 02	1.79E-14^−^
	ARIMA	2.69E + 03	1.54E-24^−^	2.71E + 03	4.24E-40^+^	4.10E + 03	7.33E-40^+^	8.56E + 03	1.36E-52^+^
	*w/l/d*	0/3/0		1/2/0		**2/1/0**		**2/1/0**	
*T*2 = 3 days	EMDE	1.60E + 03	–	2.43E + 03	–	7.31E + 02	–	3.00E + 02	–
	PR	2.79E + 03	7.00E-30^+^	2.06E + 03	1.78E-30^−^	5.43E + 03	1.16E-20^+^	1.25E + 03	5.61E-19^+^
	KRR	1.14E + 04	2.94E-47^+^	4.70E + 03	2.08E-45^+^	2.54E + 03	5.57E-13^+^	1.66E + 03	5.90E-22^+^
	ARIMA	3.45E + 03	1.57E-33^+^	2.94E + 03	4.22E-33^+^	2.16E + 03	3.54E-11^+^	1.83E + 02	2.72E-04^−^
	*w/l/d*	**3/0/0**		**2/1/0**		**3/0/0**		**2/1/0**	
*T*2 = 7 days	EMDE	1.74E + 03	–	5.55E + 03	–	2.35E + 03	–	8.39E + 02	–
	PR	8.58E + 03	2.47E-37^+^	2.53E + 03	1.74E-39^−^	1.33E + 04	1.94E-17^+^	3.46E + 03	5.95E-19^+^
	KRR	1.62E + 04	1.60E-43^+^	1.37E + 04	1.27E-47^+^	9.78E + 03	2.63E-14^+^	6.77E + 03	1.18E-25^+^
	ARIMA	4.92E + 03	5.02E-31^+^	7.02E + 03	1.59E-33^+^	5.71E + 03	2.21E-08^+^	8.99E + 02	4.15E-01^=^
	*w/l/d*	**3/0/0**		**2/1/0**		**3/0/0**		**2/0/1**	
*T*2 = 10 days	EMDE	1.55E + 03	–	7.91E + 03	–	3.56E + 03	–	1.36E + 03	–
	PR	1.92E + 04	1.53E-50^+^	8.41E + 03	4.51E-21^+^	2.17E + 04	3.29E-18^+^	6.07E + 03	5.10E-20^+^
	KRR	2.11E + 04	2.17E-51^+^	2.08E + 04	9.93E-48^+^	1.66E + 04	1.42E-15^+^	1.28E + 04	2.72E-27^+^
	ARIMA	6.68E + 03	2.53E-40^+^	1.02E + 04	1.95E-33^+^	8.68E + 03	1.70E-08^+^	1.65E + 03	1.82E-02^+^
	*w/l/d*	**3/0/0**		**3/0/0**		**3/0/0**		**3/0/0**	

In order to highlight the effectiveness of the proposed method, we have marked some experimental results that are significantly better than the comparison algorithms in bold

Second, the prediction performance of EMDE is good. In Table 3, for *T*2 = 3 days, *T*2 = 7 days, and *T*2 = 10 days, there are totally 12 (4*3) instances. Among these instances, EMDE outperforms all other data-driven methods in 6 instances and performs better than two in 6 instances. In Table 4, there also totally are 12 instances for three prediction groups. EMDE exceeds all other comparison methods in 8 instances and outperforms two in 4 instances. Therefore, we consider EMDE has the superiority of prediction ability.

Subsequently, we analyze the simulation ability and prediction ability of EMDE in the following.

1. EMDE has competitive simulation ability is acceptable. Because data-driven models are based on the actual data, they can precisely simulate the spread of the EID. However, in EMDE, due to the lack of the number of susceptible and exposed people in the epidemic mechanism, we use the simulated value of *S*(*t*) and *E*(*t*) combined with the actual data $\widetilde{I}(t)$ and $\widetilde{R}(t)$ to calculate *S*(*t* + 1), *E*(*t* + 1), *I*(*t* + 1), *R*(*t* + 1). There is a gap between the simulated data and actual data, which influences the simulation accuracy of our method. Thus, it is available that the simulation performance of EMDE cannot exceed some data-driven models, such as KRR. However, EMDE is also precise because of the existence of the epidemic mechanism that our method can capture the mechanism of epidemic transmission. Therefore, EMDE has comparable simulation capability with some data-driven models, such as PR and ARIMA.
2. We attribute the good prediction ability of EMDE to the combination of epidemic mechanism-driven and data-driven. On the one hand, the epidemic mechanism can reduce the over-focus on the changes of numerical value but not the spread of the EID. Thus, the use of the mechanism-driven method can conquer the overfit, which usually make pure data-driven methods in trouble. Moreover, the epidemic mechanism can provide the conversion relationship between different states in the spread of the EID. It increases the interpretability of epidemic spread with EMDE. On the other hand, the data-driven method can capture the unexpressed information of the mechanism. When the mechanism deviates from reality, the data-driven method can revise this deviation by the actual data. Therefore, it is reasonable that EMDE has better prediction ability than these data-driven methods.

#### Comparison experiment for the data-driven parameter optimization approach

In this section, we conduct experiments to certify the effectiveness of the data-driven parameter optimization approach in EMDE. This approach is the key to combining the mechanism-driven method and the data-driven method in EMDE. For writing convenience, we denote the EMDE model without using the data-driven parameter optimization approach as EMDE-1. All experiment settings are the same as the comparison experiment with data-driven methods for EMDE. We summarize the result in Tables 5 and 6. Since we conduct the experiment on each instance with various predicated days, there are 16 instances in each table. From Table 5, we can see EMDE significantly outperforms EMDE-1 in 12 instances, lose to EMDE-1 in 3 instances, and reaches a draw with EMDE-1 in 1 instance. In Table 6, EMDE exceeds EMDE-1 in 10 instances, loses in 3 instances, and draws 3 instances. Therefore, we can see from the experimental result that the data-driven parameter optimization approach is effective in EMDE.

**Table 5:** Comparison results for the data-driven parameter optimization (*T*1 = 7)

Predicated days	Instances/Methods	1–2 weeks		2–3 weeks		3–4 weeks		4–5 weeks	
–	–	RMSE	*P* value	RMSE	*P* value	RMSE	*P* value	RMSE	*P* value
T2 = 0 days	EMDE	3.42E + 02	–	4.64E + 02*	–	4.11E + 03*	–	2.67E + 02*	–
	EMDE-1	3.22E + 02	3.36E-01	5.18E + 02	1.16E-09	4.31E + 03	3.70E-24	2.76E + 02	9.65E-10
T2 = 3 days	EMDE	2.27E + 02*	–	7.32E + 02*	–	1.52E + 03	–	7.71E + 02*	–
	EMDE-1	4.13E + 02	5.68E-07	1.12E + 03	1.99E-15	8.45E + 02	1.56E-18	1.04E + 03	2.36E-12
T2 = 7 days	EMDE	1.86E + 03*	–	1.49E + 03*	–	5.89E + 03	–	7.94E + 02*	–
	EMDE-1	2.60E + 03	5.45E-08	2.52E + 03	5.40E-15	6.34E + 02	3.99E-33	1.53E + 03	3.90E-11
T2 = 10 days	EMDE	4.03E + 03*	–	9.05E + 03*	–	1.03E + 04	–	1.06E + 03*	–
	EMDE-1	5.09E + 03	2.41E-08	1.05E + 04	1.66E-14	2.20E + 03	2.99E-27	1.54E + 03	1.77E-07

In order to highlight the effectiveness of the proposed method, we have marked some experimental results that are significantly better than the comparison algorithms in bold

**Table 6:** Comparison results for the data-driven parameter optimization (*T*1 = 21)

Predicated days	Instances/Methods	1–4 weeks		2–5 weeks		3–6 weeks		4–7 weeks	
–	–	RMSE	*P* value	RMSE	*P* value	RMSE	*P* value	RMSE	*P* value
*T*2 = 0 days	EMDE	2.73E + 03*	–	2.60E + 03*	–	2.48E + 03*	–	2.70E + 02*	–
	EMDE-1	2.94E + 03	6.70E-14	2.67E + 03	1.05E-12	2.54E + 03	1.66E-09	2.79E + 02	2.39E-02
*T*2 = 3 days	EMDE	1.60E + 03*	–	2.43E + 03	–	7.31E + 02	–	3.00E + 02*	–
	EMDE-1	3.12E + 03	5.67E-20	2.06E + 03	3.32E-08	7.47E + 02	8.90E-01	4.82E + 02	1.47E-06
*T*2 = 7 days	EMDE	1.74E + 03*	–	5.55E + 03	–	2.35E + 03	–	8.39E + 02*	–
	EMDE-1	5.13E + 03	1.23E-22	4.84E + 03	2.53E-07	2.30E + 03	8.74E-01	1.28E + 03	6.90E-06
*T*2 = 10 days	EMDE	1.55E + 03*	–	7.91E + 03	–	3.56E + 03	–	1.36E + 03*	–
	EMDE-1	5.62E + 03	8.03E-23	6.95E + 03	1.16E-06	3.41E + 03	7.83E-01	2.00E + 03	1.95E-05

In order to highlight the effectiveness of the proposed method, we have marked some experimental results that are significantly better than the comparison algorithms in bold

It is available to believe the data-driven parameter optimization approach works. Because the actual data (**Φ**)**,** assembling the public health data and population migration data, is used not only in the objective function but also in the simulation of the EID. Since the epidemic mechanism is essentially an iterative process, if the simulation of the EID goes wrong on 1 day, the simulation of the EID will be wrong after that day. Using **Φ** in the simulation of the EID can timely revise the gap between the actual epidemic spread of the EID and the simulated epidemic spread through the epidemic mechanism. In other words, this approach drives the simulation of the EID as close to the actual spread as possible. However, the actual data of the susceptible people and the exposed people are unavailable, which may affect the performance of our approach. Thus, it is also reasonable that EMDE loses to or draws with EMDE-1 in fewer instances.

#### Comparison experiment with numerical optimization methods

In the third experiment, we compare the swarm-optimizer-assisted simulation module (SSM) with two numerical optimization methods to prove the validity, which are traditionally used in parameter optimization for epidemic mechanisms. A nonlinear least-squares curve-fitting with 4^th^ order Runge–Kutta method (NLSRK) [15], and a least square and Markov Chain Monte Carlo (LSMCMC) method [43] are used. Two groups of simulation experiments are conducted, where the simulation days, respectively, are 7 days and 21 days. The results are shown in Tables 7 and .

**Table 7:** Comparison results with numerical methods for simulation days being 7 days (*T*1 = 7)

Instances/Methods	1–2 weeks		2–3 weeks		3–4 weeks		4–5 weeks	
–	RMSE	*P* value	RMSE	*P* value	RMSE	*P* value	RMSE	*P* value
SSM	3.42E + 02	–	4.64E + 02	–	4.11E + 03	–	2.67E + 02	–
NLSRK	4.05E + 02	4.84E-03^+^	1.31E + 03	3.77E-43^+^	1.38E + 04	2.49E-69^+^	1.86E + 04	9.62E-73^+^
LSMCMC	9.50E + 04	1.63E-26^+^	4.72E + 04	9.97E-49^+^	1.09E + 04	2.93E-56^+^	1.04E + 05	1.58E-52^+^
w/l/d	2/0/0		2/0/0		2/0/0		2/0/0	

In Tables 7 and 8, SSM outperforms LSMCMC in all instances, and only loses to NLSRK in one instance. Thus, we recognize SSM has effectiveness compared with numerical optimization methods. This conclusion is reasonable and we analyze it in the following:

1. The swarm optimizer is used in SSM to optimize the parameters, which has good performance in solving optimization problem.
2. Moreover, the LLSO algorithm has good performance in the high-dimension problem, which satisfies the requirement of our model. However, when the number of parameters increases, the traditional numerical method is difficult to work well.

**Table 8:** Comparison results with numerical methods for simulation days being 21 days (*T*1 = 21)

Instances/Methods	1–4 weeks		2–5 weeks		3–6 weeks		4–7 weeks	
–	RMSE	*P* value	RMSE	*P* value	RMSE	*P* value	RMSE	*p* value
SSM	2.73E + 03	–	2.60E + 03	–	2.48E + 03	–	2.70E + 02	–
NLSRK	2.64E + 03	7.07E-31^−^	4.37E + 03	3.05E-63^+^	5.77E + 03	9.96E-46^+^	9.04E + 03	4.70E-53
LSMCMC	1.65E + 04	1.58E-46^+^	7.24E + 05	1.85E-52^+^	2.57E + 04	2.14E-27^+^	9.00E + 03	6.74E-39
w/l/d	1/1/0		2/0/0		2/0/0		2/0/0	

#### Comparison experiment with other particle swarm optimizers

**Fig. 4:**
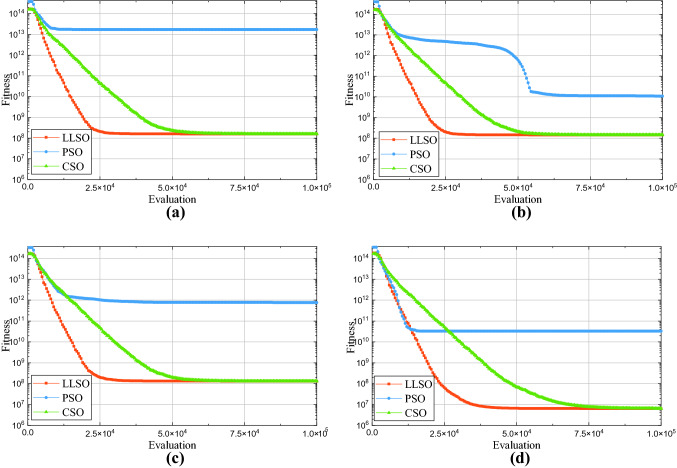
Comparison results with PSO and CSO in different instances. **a** 1–4 weeks **b** 2–5 weeks **c** 3–6 weeks **d** 4–7 weeks

In this experiment, we compare LLSO with PSO and competitive swarm optimizer (CSO) [44] under the same evaluation times. Apart from the swarm optimizer used in the swarm-optimizer-assisted simulation module being different, all other experimental settings are the same, where *T*1 = 21, *T*2 = 0, *T*3 = 3. To better verify the advantages of LLSO, we conduct the comparison on four instances. The result is shown in Fig. 4. In the comparison result, LLSO outperforms PSO and CSO in all instances. Although CSO has almost the same convergence result as LLSO with enough evaluation times, LLSO is faster than CSO to reach convergence. Moreover, whether convergence speed or convergence result, PSO is not good as LLSO and CSO. Therefore, compared with PSO and CSO, LLSO has the advantage of being the swarm optimizer in the swarm-optimizer-assisted simulation module.

## Discussion

- (1). Although EMDE only has the competitive simulation ability compared with data-driven methods, EMDE shows good prediction ability. We contribute it to the combination of the epidemic mechanism-driven method and the data-driven method. With better prediction ability, EMDE can help the government and medical institutions take more precise epidemic prevention measures.
- (2). From the experiment of studying the effectiveness of the data-driven parameter optimization approach, we can see that this approach is effective in EMDE. Since the approach is the key to combining mechanism-driven and data-driven methods, the validity of the approach demonstrates the effective combination of mechanism-driven and data-driven methods.
- (3). Compared with numerical optimization methods and other particle swarm optimizers, SSM used LLSO shows superiorities in the simulation and prediction of the spread of the EID in the last two experiments.

## Conclusions

First, we construct an EMDE model for the EID. In EMDE, due to the unignored influence of the population migration on the spread of the EID, the population migration is considered into a standard SEIR mechanism to simulate the spread process of the EID. Second, we assemble epidemic data and population data to construct a data-driven approach in EMDE to optimize parameters for the improved mechanism. Third, under the improved model, a swarm-optimizer-assisted simulation and prediction method is proposed, which contains two modules. In the first module, the LLSO algorithm assist EMDE optimize the required parameters. In the second module, based on the epidemic mechanism with population migration and optimized parameters, the EID spread in the next few days is predicted. Moreover, we compare with our work with data-driven methods and numerical optimization methods to validate the effectiveness of EMDE and SSM.

In the future, the control of EIDs in large-scale networks will remain a difficult problem. Studying large-scale network propagation [45], tracking important nodes in large-scale networks [46], extracting significant network structure [47], may help solve this problem.

## Data Availability

All data are collected from https://qianxi.baidu.com/ and github.com/ BlankerL/DXY-COVID-19-Data.

## References

[1] Priyadarsini SL, Suresh M, Huisingh D (2020). What can we learn from previous pandemics to reduce the frequency of emerging infectious diseases like COVID-19?. Glob Transitions.

[2] WHO (2021) Weekly Operational Update on COVID-19. Emerg. Situational Updat. 1–10

[3] Sabin NS, Calliope AS, Simpson SV (2020). Implications of human activities for (re)emerging infectious diseases, including COVID-19. J Physiol Anthropol.

[4] Metcalf CJE, Lessler J (2017). Opportunities and challenges in modeling emerging infectious diseases. Science.

[5] Kermack WO, McKendrick AG (1991). Contributions to the mathematical theory of epidemics—I. Bull Math Biol.

[6] Li MY, Muldowney JS (1995). Global stability for the SEIR model in epidemiology. Math Biosci.

[7] Hou C, Chen J, Zhou Y (2020). The effectiveness of quarantine of Wuhan city against the Corona Virus Disease 2019 (COVID-19): a well-mixed SEIR model analysis. J Med Virol.

[8] Cai LM, Li XZ (2009). Analysis of a SEIV epidemic model with a nonlinear incidence rate. Appl Math Model.

[9] Zhou X, Cui J (2011). Analysis of stability and bifurcation for an SEIV epidemic model with vaccination and nonlinear incidence rate. Nonlinear Dyn.

[10] Pare PE, Liu J, Beck CL (2020). Analysis, estimation, and validation of discrete-time epidemic processes. IEEE Trans Control Syst Technol.

[11] Wang Y, Chakrabarti D, Wang C, Faloutsos C (2003) Epidemic spreading in real networks: An eigenvalue viewpoint. In: Proceedings of the IEEE Symposium on Reliable Distributed Systems. pp 25–34

[12] Zu J, Li M, Li Z, et al (2020) Epidemic trend and transmission risk of SARS-CoV-2 after Government Intervention in the Mainland of China: a mathematical model study. SSRN Electron J 1–31

[13] Carcione JM, Santos JE, Bagaini C, Ba J (2020). A simulation of a COVID-19 epidemic based on a deterministic SEIR model. Front Public Heal.

[14] Rezapour S, Mohammadi H, Samei ME (2020). SEIR epidemic model for COVID-19 transmission by Caputo derivative of fractional order. Adv Differ Equations.

[15] López L, Rodó X (2021). A modified SEIR model to predict the COVID-19 outbreak in Spain and Italy: simulating control scenarios and multi-scale epidemics. Results Phys.

[16] Annas S, IsbarPratama M, Rifandi M (2020). Stability analysis and numerical simulation of SEIR model for pandemic COVID-19 spread in Indonesia. Chaos, Solitons Fractals.

[17] Wieczorek M, Siłka J, Woźniak M (2020). Neural network powered COVID-19 spread forecasting model. Chaos, Solitons Fractals.

[18] Rustam F, Reshi AA, Mehmood A (2020). COVID-19 future forecasting using supervised machine learning models. IEEE Access.

[19] Hu Z, Ge Q, Li S, Xiong M (2020). Artificial Intelligence Forecasting of Covid-19 in China. Int J Educ Excell.

[20] Hazarika BB, Gupta D (2020). Modelling and forecasting of COVID-19 spread using wavelet-coupled random vector functional link networks. Appl Soft Comput J.

[21] Dogan O, Tiwari S, Jabbar MA, Guggari S (2021). A systematic review on AI/ML approaches against COVID-19 outbreak. Complex Intell Syst.

[22] Elmousalami HH, Hassanien AE (2020) Day level forecasting for coronavirus disease (COVID-19) spread: analysis, modeling and recommendations. arXiv. 10.48550/arXiv.2003.07778

[23] Maleki M, Mahmoudi MR, Wraith D, Pho K-H (2020). Time series modelling to forecast the confirmed and recovered cases of COVID-19. Travel Med Infect Dis.

[24] Tandon H, Ranjan P, Chakraborty T, Suhag V (2020) Coronavirus (COVID-19): ARIMA based time-series analysis to forecast near future. ArXiv. 10.48550/ARXIV.2004.07859

[25] Jiang-ning L, Xian-liang S, An-qiang H (2021). Forecasting emergency medicine reserve demand with a novel decomposition-ensemble methodology. Complex Intell Syst.

[26] Yang Z, Zeng Z, Wang K (2020). Modified SEIR and AI prediction of the epidemics trend of COVID-19 in China under public health interventions. J Thorac Dis.

[27] Feng S, Feng Z, Ling C (2021). Prediction of the COVID-19 epidemic trends based on SEIR and AI models. PLoS ONE.

[28] Fu X, Small M, Walker DM, Zhang H (2008). Epidemic dynamics on scale-free networks with piecewise linear infectivity and immunization. Phys Rev E Stat Nonlinear Soft Matter Phys.

[29] Korobeinikov A (2004). Lyapunov functions and global properties for SEIR and SEIS epidemic models. Math Med Biol.

[30] Eberhart R, Kennedy J (1995) New optimizer using particle swarm theory. In: Proceedings of the International Symposium on Micro Machine and Human Science. pp 39–43

[31] Zhan C, Tse CK, Fu Y (2020). Modeling and prediction of the 2019 coronavirus disease spreading in China incorporating human migration data. PLoS ONE.

[32] Busenberg SN, Travis CC (1983). Epidemic models with spatial spread due to population migration. J Math Biol.

[33] Chen ZL, Zhang Q, Lu Y (2020). Distribution of the COVID-19 epidemic and correlation with population emigration from Wuhan, China. Chin Med J (Engl).

[34] 5 million-plus leave Wuhan. 5 million-plus leave Wuhan

[35] Du Z, Wang L, Cauchemez S (2020). Risk for transportation of coronavirus disease from Wuhan to other cities in China. Emerg Infect Dis.

[36] Dang Q, Gao W, Gong M (2022). Multiobjective multitasking optimization assisted by multidirectional prediction method. Complex Intell Syst.

[37] Zhao T-F, Chen W-N, Liew AW-C (2021). A binary particle swarm optimizer with priority planning and hierarchical learning for networked epidemic control. IEEE Trans Syst Man, Cybern Syst.

[38] Liang JJ, Qin AK, Suganthan PN, Baskar S (2006). Comprehensive learning particle swarm optimizer for global optimization of multimodal functions. IEEE Trans Evol Comput.

[39] Wei FF, Chen WN, Yang Q (2021). A classifier-assisted level-based learning swarm optimizer for expensive optimization. IEEE Trans Evol Comput.

[40] Yu Y, Xu Y, Wang F (2021). Adsorption control of a pipeline robot based on improved PSO algorithm. Complex Intell Syst.

[41] Yang Q, Chen W, Deng JD (2018). A level-based learning swarm optimizer for large-scale optimization. IEEE Trans Evol Comput.

[42] Chen Q, Yan J, Huang H, Zhang X (2021). Correlation of the epidemic spread of COVID-19 and urban population migration in the major cities of Hubei Province, China. Transp Saf Environ.

[43] Zu J, Li ML, Li ZF (2020). Transmission patterns of COVID-19 in the mainland of China and the efficacy of different control strategies: a data- And model-driven study. Infect Dis Poverty.

[44] Cheng R, Jin Y (2015). A competitive swarm optimizer for large scale optimization. IEEE Trans Cybern.

[45] Chen WN, Tan DZ, Yang Q (2020). Ant colony optimization for the control of pollutant spreading on social networks. IEEE Trans Cybern.

[46] Liu S, Liu D, Srivastava G (2021). Overview and methods of correlation filter algorithms in object tracking. Complex Intell Syst.

[47] Teng X, Liu J, Li M (2021). Overlapping community detection in directed and undirected attributed networks using a multiobjective evolutionary algorithm. IEEE Trans Cybern.

